# Analysis of the Molecular Mechanism of Parturition and the Establishment of a Safe Obstetrical Care System - Secondary Publication

**DOI:** 10.31662/jmaj.2025-0099

**Published:** 2025-06-13

**Authors:** Tadashi Kimura

**Affiliations:** 1 Sakai City Hospital Organization, Osaka, Japan

**Keywords:** Onset of Labor, Preeclampsia, Maternal near miss, Japan Environment and Children’s Study (JECS), National survey on environment and children’s health by the Ministry of the Environment

## Abstract

The article presents the research and social implementation efforts undertaken by my colleagues and me at the Department of Obstetrics and Gynecology, Osaka University Graduate School of Medicine. We cloned the human oxytocin receptor using molecular biology techniques and investigated its transcriptional regulatory mechanisms in the human uterus during parturition. Additionally, we adapted a mouse placenta-specific gene expression system to develop a preeclampsia model and explored potential therapeutic strategies. Our research also focused on improving surgical techniques for peripartum hysterectomy in cases of critical obstetric hemorrhage and vasa praevia with placenta praevia, which may also lead to sudden fetal death during parturition. Concurrently, based on epidemiological data, we adopted a social approach strengthening obstetric care teams and identifying key issues within Japan’s delivery system. Furthermore, we developed a web-based postpartum care procedure. I hope that research and practices aimed at enhancing maternal safety and well-being will continue to progress further in the future.

## Introduction

Heavy bleeding during childbirth/obstetrical situations and preeclampsia (a hypertensive disorder during pregnancy), the two leading causes of maternal death in the 1950s and 1960s, continue to pose serious medical challenges. Elucidating the molecular mechanism of uterine contraction and the onset of labor via oxytocin (OT) remains a crucial issue in obstetrics. Analyzing the pathophysiology of these conditions using molecular biology techniques and improving practical procedures are critical for advancing obstetrical care. The Japanese perinatal care system differs significantly from other countries with high medical resources, making an epidemiological survey of our system essential. Additionally, developing web-based tools to enhance maternal safety within the obstetric care system is an important area of focus. This report outlines the research and social implementation efforts my colleagues and I conducted at the Department of Obstetrics and Gynecology, Osaka University Graduate School of Medicine.

## From the Molecular Analysis of Childbirth- and Pregnancy-related Complications to Interventions against Maternal Near-miss Cases

OT (a peptide hormone composed of nine amino acids) has been widely used since the 1950s to induce and augment labor and treat postpartum hemorrhage. Maternal OT levels in blood do not change before or after the onset of labor. Additionally, it is known that the uterine contractile response to the same dose of OT, i.e., uterine sensitivity to OT, varies significantly depending on the gestational week and between individuals, even at the same gestational stages ^(1)^. To elucidate the molecular mechanism underlying uterine sensitivity to OT, we cloned the human OT receptor (OTR) complementary deoxyribonucleic acid (DNA) using the Xenopus laevis oocyte expression system ^(2)^. OTR messenger ribonucleic acid levels in the myometrium at parturition were more than 300-fold higher than those in the myometrium of a non-pregnant uterus ^(3)^ and increased in the decidua as parturition progressed ^(4)^. OT is also produced locally in the decidual membrane. Therefore, in addition to its endocrine role, OT has an autocrine effect in the decidual membrane during parturition. The gene is located on the short arm of chromosome 3 ^(5)^. We cloned the DNA-binding protein hMafF, which binds to the upstream region of the OTR gene and is upregulated in the myometrium during parturition ^(6)^. However, hMafF lacks a transactivation domain, suggesting that other transcription modulators may be involved in regulating this gene. Notably, OTR-deficient mice conceived and underwent delivery typically, indicating that the OT-OTR system does not play a central role in the initiation and progression of parturition. However, the pups born to OTR-deficient mothers starved to death because the mothers were unable to eject milk. Additionally, OTR-deficient mice nursed by foster mothers exhibited excessive aggression (observed in males) and deficient maternal behavior (observed in females), providing the basis for later studies regarding the role of central OT action ^(7)^.

Preeclampsia was the second leading cause of maternal mortality until the 1960s and remains the second leading cause of maternal near miss (obstetric conditions requiring intensive care to prevent maternal death), occurring in approximately 5% of pregnant women (including non-severe cases). One known cause of preeclampsia is the overproduction of soluble vascular endothelial growth factor receptor s-Flt-1 in the placenta. The only effective treatment for preeclampsia remains termination of pregnancy (placental delivery). Using a technique to express a gene specifically in the placenta, we created a mouse model of preeclampsia by overexpressing human s-Flt-1. In this model, maternal administration of pravastatin early in pregnancy prevented the onset of preeclampsia, providing insights for clinical trials in which pravastatin is administered during pregnancy to prevent or treat this condition ^(8)^.

The causes of massive uterine hemorrhage that lead to maternal near-miss events in clinical practice include uterine atony, placenta previa, placenta accreta and placenta percreta sequence, and placental abruption. In addition, vasa praevia, in which fetal vessels pass over the amniotic membrane covering the uterine os and are often associated with placenta previa, causes fetal hemorrhage and death immediately after rupture of membranes. Compression sutures help control uterine hemorrhage; however, it is difficult for a curved needle to penetrate the thick uterine wall. We developed a straight blunt needle with an absorbable thread to simplify the compression suture procedure ^(9)^. This procedure requires lifting the uterus out of the pelvic cavity; however, if there is an adhesion between the posterior surface of the uterus and the pelvic wall, lifting the uterus without paying attention to the adhesion may exacerbate the condition, leading to heavy bleeding from the adhesive surface. We developed an index to predict the presence of such adhesions based on the angle of the uterine cervical canal and the posterior uterine wall observed on MRI images taken to differentiate between placenta previa and placenta accreta, which are conditions that often require compression sutures for hemostasis ^(10)^. We also reported that in cases involving vasa previa, cesarean section performed without rupturing the membranes until just before fetal delivery can be safely conducted ^(11)^.

## Changes in Osaka’s Perinatal Care System to Manage Maternal Near-miss Events

A system in which a small team of doctors in the Department of Obstetrics and Gynecology manages the delivery ward at night and on holidays would not comply with the Labor Standards Act. This obstetric and gynecologic service was discontinued at Izumisano Municipal Hospital and Kaizuka City Hospital–affiliated with Osaka University–in the southern part of Osaka Prefecture in 2006. Izumisano Municipal Hospital (currently Rinku General Medical Centre) has an Advanced Emergency and Critical Care Centre and a Neonatal Intensive Care Unit (NICU). I engaged in extensive negotiation with the executives of both hospitals and local governments, and they agreed to renovate the obstetric and gynecologic system in both hospitals. Under the new arrangement, the Izumisano Municipal Hospital became responsible for deliveries and night-time obstetric and gynecologic emergencies, while Kaizuka City Hospital was responsible for scheduled gynecologic surgeries. Doctors from both hospitals were required to work as a single team. Subsequently, two neighboring cities and three towns joined the initiative, establishing the Senshu Regional Maternal and Child Medical Centre and the Senshu Regional Gynaecological Medical Centre, which began operations in April 2008. By centralizing delivery facilities, a high level of medical care can now be provided without delay. In collaboration with the Institute of Social and Economic Research at Osaka University, we conducted an analysis comparing the economic effects of centralizing delivery services. The study assessed the benefits received by residents of childbearing and child-rearing age against the costs incurred in taxes. The results showed that the benefits exceeded the costs ^(12)^. We initiated treating maternal near-miss cases in the Emergency Medicine Centre, initially involving staff from the Traumatology and Acute Critical Medicine and the Obstetrics and Gynacology departments at Osaka University Hospital in the early 2000s. A similar collaboration began at the Senshu Regional Maternal and Child Medical Centre following the centralization of obstetrical service. Initially, many doctors in Japan were concerned about overburdening the Emergency Medicine Centre; however, maternal near miss accounted for only 1% of the total patients treated in the center, and their length of stay was short, alleviating these concerns. As a result, Osaka Prefecture has accredited nine (now ten) facilities where advanced emergency centers and obstetric departments work together as part of a project to manage maternal near-miss cases, i.e., seriously ill pregnant women. Their treatment outcomes are recorded annually. Additionally, basic information contributing to maternal safety is now collected. For instance, it has been determined that more than one in 200 pregnant women (100,000:500, a rate comparable to the maternal mortality rate in Sub-Saharan Africa at the end of the 20th century) experience a maternal near miss every year ^(13)^.

## Lessons Learned from Large-scale Epidemiological Surveys

The only large-scale perinatal database in Japan is maintained by the Japan Society of Obstetrics and Gynecology (which registers approximately one-third of all births each year). However, it does not contain information on children beyond the early neonatal period (one week after birth). The Japan Environment and Children’s Study (JECS)―a national survey on environment and children’s health by the Ministry of the Environment―established a cohort of 100,000 pregnant women, their partners, and their newborns nationwide over two years (2011-2013). The children’s developments are followed for more than 15 years after birth. At that time, we believed this cohort would be the only one to link data between pregnant women and their children. We collaborated on this project with Professor Hiroyasu Iso from the Department of Public Health at our university and recruited over 8,000 pregnant women in the southern area of Osaka, with the cooperation of the Obstetrical and Gynecological Society of Osaka.

Most birth facilities in Japan lack neonatal care units. Newborns that are delivered under poor conditions at such facilities are transferred to secondary/tertiary facilities with NICUs to save their lives. Although Japan has a significantly low neonatal mortality rate (fewer than one per 1,000 live births), the long-term prognosis for infants remains uncertain. We compared neurodevelopmental outcomes (assessed using ASQ-3) at three years of age among children registered in the JECS. This included those born in poor conditions and transferred and cared for in an NICU versus those treated in an in-house NICU without transfer. The results indicated that children transferred neonatally were more likely to exhibit developmental issues, and this finding remained consistent when considering children admitted for longer than seven days in NICU and those with an Apgar score <7 ^(14)^. These findings suggest that delay and other factors associated with neonatal transfer may impact future neurodevelopmental outcomes.

Given the dispersion of small birth facilities across the country, it is crucial to analyze the maternal risks associated with childbirth in advance. This analysis aims to encourage high-risk pregnant women to deliver in secondary or tertiary facilities capable of providing comprehensive medical care. Within the JECS cohort, pregnant women were surveyed about their autistic tendencies during pregnancy using a self-administered questionnaire (Autism-Spectrum Quotient-Japanese version 10). Women with a high autistic tendency score had increased odds of preterm birth (<32 weeks, 32-36 weeks) and giving birth to low birth weight babies compared to those with lower scores ^(15)^.

## Web-based Assessment of Mothers for Addressing Future Parenting/Nursing Challenges

Using web-based (online) interviews represents a significant advancement for future healthcare. We focused on postpartum parenting/nursing difficulties and conducted a cross-sectional online survey using the Comprehensive Scale for Parenting Resilience and Adaptation, developed in collaboration with the School of Human Sciences at Osaka University. Through collaboration with RIKEN, we successfully applied machine-learning techniques to identify mothers experiencing significant parenting/nursing difficulties ^(16)^. These results have been validated at the municipal level, and demonstration experiments are underway to support individuals facing more significant difficulties with childcare. These experiments include peer online meetings with experts and targeted health counseling.

## Conclusion

This article outlines the research I conducted in collaboration with my colleagues. The research ranges from analyzing molecular mechanisms of parturition to clinical and social medical implications, all aimed at fostering a healthcare system that ensures safe and secure childbirth. I would like to express my deepest gratitude to the many collaborators whose dedication has been instrumental in achieving this goal. We hope that, in this era of sharply declining fertility rates in Japan, researchers will continue to advance their efforts toward safer and more reassuring experiences associated with pregnancy, childbirth, and parenting.

## Article Information

This article is based on the study, which received the Medical Award of The Japan Medical Association in 2024. This is a revised English version of the article originally published in Japanese in the Journal of the Japan Medical Association 2025;153(10):1115-7 ^(17)^. The original version is available at https://med.or.jp/cme/jjma/newmag/pdf/153101115.pdf. Only members of the Japan Medical Association are able to access it.

### Conflicts of Interest

None
